# Ameliorating Effect of Fermented *Perilla frutescens* on Sleep Deprivation-Induced Cognitive Impairment Through Antioxidant and BDNF Signaling in Mice

**DOI:** 10.3390/nu16234224

**Published:** 2024-12-06

**Authors:** Chae-Ryeong Seo, Bo Kyung Lee, Hye Jin Jee, Jae Ryeong Yoo, Chul-Kyu Lee, Jin Wook Park, Yi-Sook Jung

**Affiliations:** 1 Department of Pharmacy, Ajou University, Suwon 16499, Republic of Korea; wb0720@ajou.ac.kr (C.-R.S.); pfiffer@ajou.ac.kr (B.K.L.); hjjee@ajou.ac.kr (H.J.J.); jryoo@ajou.ac.kr (J.R.Y.); 2 Headquarters of New Drug Development Support, Corestemchemon Inc., 15 F, Gyeonggi Bio Center, Suwon 16229, Republic of Korea; cklee@csco.co.kr (C.-K.L.); jiwpark@csco.co.kr (J.W.P.); 3 Research Institute of Pharmaceutical Sciences and Technology, Ajou University, Suwon 16499, Republic of Korea

**Keywords:** *Perilla frutescens*, fermentation, cognitive impairment, sleep-deprivation, oxidative stress, brain-derived neurotrophic factor

## Abstract

**Background:** Adequate sleep is essential for maintaining cognitive function, as evidenced by literature. *Perilla frutescens* var. *acuta Kudo* (PF) is a traditional medicinal herb reported to improve vascular cognitive impairment and induce sedation. However, the effects of PF on cognitive impairment caused by sleep deprivation (SD) have not yet been evaluated. This study aims to evaluate the effects of fermented PF (FPF) and its underlying mechanisms in a model of SD-induced cognitive impairment. **Methods:** Mice were subjected to SD to establish cognitive impairment, and FPF was administered once daily for 3 days. Cognitive performance was assessed using Y-maze and passive avoidance tests, followed by molecular mechanisms analyses. **Results:** FPF treatment improved SD-induced cognitive impairment, as evidenced by increased spontaneous alternation and extended latency time. Histological analysis revealed that SD impaired the hippocampus, and this impairment was alleviated by FPF treatment. FPF demonstrated antioxidant activity by increasing glutathione levels and decreasing malondialdehyde levels. Furthermore, the decreased levels of brain-derived neurotrophic factor (BDNF) observed in sleep-deprived mice were restored with FPF treatment. FPF also enhanced the phosphorylation of tropomyosin receptor kinase B, extracellular signal-regulated kinase, and cAMP response element-binding protein. **Conclusions:** These results indicate that FPF may have beneficial effects on SD-induced cognitive impairment by protecting against oxidative stress and increasing BDNF expression.

## 1. Introduction

In recent decades, growing evidence has suggested a link between cognitive function and sleep [1]. Sleep is an essential biological response for maintaining physical and mental health [2]. It can help restore the central nervous system by removing the waste accumulated while awake [3]. It can also embed learned information into long-term memory through active system consolidation processes [4]. Adequate quantity and quality of sleep are crucial for maintaining brain function [5]. However, more than one-third of adults regularly fail to obtain sufficient sleep, raising concerns worldwide. It has been reported that SD tends to increase with age and is more prevalent in women than in men, likely due to hormonal changes [6]. Moreover, insufficient sleep is not only associated with a higher risk of obesity, cardiovascular diseases, and type 2 diabetes but also contributes to cognitive impairments, including reduced attention, learning difficulties, and memory deficits [7]. Studies conducted on healthy humans reported that SD was associated with the increased amyloid burden in the brain [8], and individuals with sleep disorders had a higher risk of developing cognitive impairment or Alzheimer’s disease (AD) or both compared to individuals without sleep disorders [9]. Another follow-up study showed that middle-aged people with persistent short-term sleep had an increased risk of dementia compared with those having a normal sleep duration [10]. In modern society, an increasing number of people do not sleep sufficiently. Given the negative effects of insufficient sleep, SD is a global public health concern [11]. Therefore, it is necessary to understand how SD impairs cognition and to identify strategies for its treatment and prevention.

The mechanisms underlying SD-induced cognitive impairment remain unclear, but oxidative stress in the hippocampus has been assumed to be a potential mechanism [12]. The hippocampus is a major brain region involved in learning, memory, and emotional integration [13]. The brain is particularly susceptible to oxidative stress owing to its relatively low antioxidant content, high oxygen consumption, and high lipid content [14]. In fact, sleep-deprived mice show reactive oxygen species (ROS) accumulation and endogenous antioxidant depletion in the hippocampus [15,16]. In addition, SD can increase glutamate levels in the hippocampus, which has a serious impact on the neuronal damage by causing ROS accumulation [17,18]. As a result, the oxidative stress in the hippocampus causes various pathological conditions and increases the risk of neurological disorders [19]. Numerous studies have proposed that the reduction in brain-derived neurotrophic factor (BDNF) expression is another mechanism of SD-induced cognitive impairment [20,21]. BDNF is a critical neurotrophin that supports central nervous system development [22,23]. It regulates neurophysiological processes such as neuroprotection and synaptic plasticity through signaling cascades induced by binding to its receptor, tropomyosin receptor kinase B (TrkB) [23,24]. In humans, long-term potentiation (LTP)-like plasticity is reduced in individuals undergoing SD, and this reduction is associated with lower plasma BDNF levels compared to healthy controls [25]. Furthermore, studies in various rodent models have demonstrated that higher levels of BDNF are correlated with improvements in cognitive impairment induced by SD [21,26]. Additionally, the pre-treatment with ANA12, a TrkB antagonist, has been reported to block BDNF signaling and exacerbate cognitive impairments [27], further suggesting the BDNF signaling through TrkB is closely linked to cognitive dysfunction.

*Perilla frutescens* var. *acuta Kudo* (PF) is an herbal plant belonging to the Lamiaceae family that is cultivated worldwide, especially in East Asian countries. Many pharmacological studies have reported that PF possesses abundant biological activities, such as antioxidant, anticancer, anti-inflammatory, and antidepressant activities [28,29,30,31]. Therefore, PF is used in the form of traditional medicines, spices, and foods [32,33]. According to recent studies, the PF leaf extract ameliorates cognitive impairment and hippocampal neuronal death in a vascular dementia model [34]. Another study reported that PF essential oil has sedative and hypnotic effects [35]. Based on these reports, we hypothesized that PF may affect the cognitive impairment induced by SD. Recently, fermentation has attracted attention because it can improve the efficacy and stability of biologically active components in microorganisms [36]. PF has been considered a great source for fermentation, and fermentation of PF increases the biosynthesis of metabolites, including flavonoids, phenols, and fatty acids [37]. However, there are no reports on the effects of fermented PF (FPF) on cognitive impairment. In this study, we aimed to evaluate the effects of FPF on SD-induced cognitive impairment and investigate the underlying mechanisms.

## 2. Materials and Methods

### 2.1. Reagents

PF was purchased from Farm Herbnara (Pyeongchang, Republic of Korea). L-Glutamic acid (Glutamate), 2,2-diphenyl-1-picrylhydrazyl (DPPH), 2,2′-azinobis (3-ethylbenzothiazoline-6-sulfonic acid (ABTS), and *N*-[2-[(hexahydro-2-oxo-1H-azepin-3-yl)amino]carbonyl]phenyl-benzo [b] thiophene-2-carboxamide (ANA12) were purchased from Sigma-Aldrich (Saint Louis, MO, USA). The glutathione assay kit was purchased from Cayman Chemical (Ann Arbor, MI, USA). Anti-BDNF, anti-TrkB, anti-p-cAMP response element-binding protein (CREB), anti-CREB, anti-p-extracellular signal-regulated kinase (ERK), anti-ERK, and anti-gylceraldehyde-3-phosphate dehydrogenase (GAPDH) antibodies were purchased from Cell Signaling Technology (Danvers, MA, USA). The anti-TrkB antibody was purchased from Abcam (Cambridge, MA, USA). 2,7-dichlorodihydro fluorescent diacetate (H_2_DCFDA) was purchased from Thermo Fisher Scientific (Waltham, MA, USA). 6-Hydroxy-2,5,7,8-tetramethylchroman-2-carboxylic acid (Trolox) was purchased from Tocris Bioscience (Ellisville, MO, USA). All other chemical reagents were purchased from Sigma-Aldrich and were of analytical or high-performance liquid chromatography grade.

### 2.2. Preparation of FPF

Dried leaves of PF (15 g) were extracted in distilled water (1% *w*/*v*) at 80 °C for 24 h and filtered. The PF extracts were fermented with *Bacillus subtilis* KACC 10,111 for 3 days at 30 °C and 130 rpm. The fermented broth was sterilized at 121 °C and filtered with a 0.45 μm filter membrane. Finally, the FPF was freeze-dried, and 4.05 g (27%) of sample was obtained.

### 2.3. Animals

**Figure 1:**
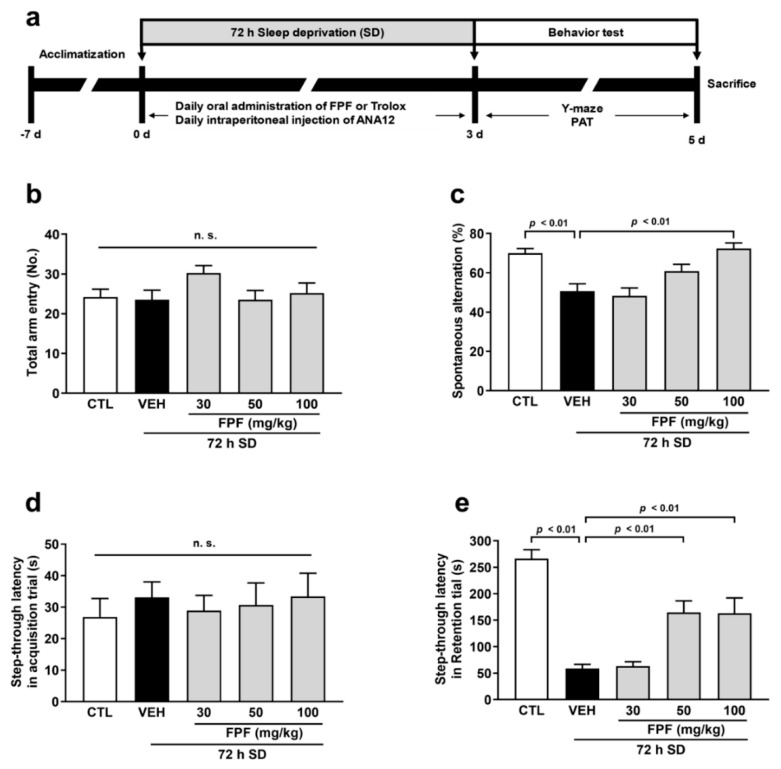
Effect of FPF on cognitive impairment in sleep-deprived mice. (**a**) The schematic experimental procedure. The FPF (30, 50, and 100 mg/kg, p.o) was administered to sleep-deprived mice once daily for 3 days. After the administration, behavioral tests were performed. (**b**) The total arm entries (one-way ANOVA: F_(4, 44)_ = 1.475, *p* = 0.2259) and (**c**) the spontaneous alternation (one-way ANOVA: F_(4, 44)_ = 10.59, *p* < 0.0001) in the Y-maze test. (**d**) The step-through latency in acquisition trial (one-way ANOVA: F_(4, 44)_ = 0.2056, *p* = 0.9340) and (**e**) the step-through latency in retention trial (one-way ANOVA: F_(4, 44)_ = 20.34, *p* < 0.0001) of the PAT. Data are presented as the mean ± SEM (*n* ≤ 10). CTL, control; VEH, vehicle; SD, sleep deprivation; FPF, fermented *Perilla frutescens*; PAT, passive avoidance test; n.s., not significant; ANOVA, analysis of variance; SEM, standard error of mean.

Seven-week-old C57BL/6N male mice (20–24 g) were purchased from Orient Bio Inc. (Seongnam, Republic of Korea). The animals were kept at 23 ± 1 °C and 60 ± 10% humidity and provided access to water ad libitum. The light and dark cycles in the room were altered every 12 h (lights on at 9:00 and off at 21:00). The animals were allowed to acclimate for at least 1 week prior to the experiment. All experimental procedures were approved by the Institutional Animal Care and Use Committee of Ajou University (Approval Number 2021-0077). The animals were randomly divided into the following groups: control, SD, SD + FPF (30, 50, and 100 mg/kg, p.o.), SD + Trolox (50 mg/kg, p.o.), and SD + ANA12 (0.5 mg/kg, i.p.) + FPF (100 mg/kg, p.o.). Trolox functions as an antioxidant, scavenging free radicals, while ANA12, a TrkB antagonist, inhibits TrkB signaling by preventing BDNF from binding to the TrkB. Therefore, we used these two drugs to investigate how FPF affects ROS and BDNF signaling pathways. Additionally, the concentration of the two drugs was determined based on previous studies [38,39]. Figure 1a shows the experimental procedure. The number of animals per group was limited to 10 for each experiment, and since the amount of samples and methodologies required for each experiment varied, the total number of animals used across all experiments exceeded 250. Each group was subjected to SD for 72 h, and the control group was left in the home cage. The FPF or Trolox groups were orally administered once daily for 3 days. ANA12 was intraperitoneally administered 15 min before FPF treatment for 3 days. The control and vehicle groups were administered the equal volume of the solvent for each drug. Furthermore, in all animals that received oral or intraperitoneal administration, the administration itself did not affect the phenotype of the disease model, thus ruling out potential confounding factors in the experimental results. All drugs were freshly prepared for daily use. FPF and Trolox were dissolved in saline and 40% polyethylene glycol, respectively. ANA12 was dissolved in 1.25% dimethyl sulfoxide (DMSO) with 1% Tween 80 in saline. After three days, the behavioral tests were performed. All animal experiments were conducted between 9:00 and 12:00.

### 2.4. SD-Induced Cognitive Impairment Mice Model

The SD-induced cognitive impairment model was instituted by disrupted sleep using the modified multiple-platform method [40]. The tanks (42 × 26 × 18 cm) contained eight cylindrical acrylic platforms (3 cm in diameter each), and each platform was constructed at a distance of 5 cm. This distance was chosen for free movement and the jumping of the mice on the platform. The mice were placed in a tank filled with water (1 cm below the platform surface) without touching the water for 72 h. During the SD period, mice had ad libitum access to water and food, which were replaced once daily.

### 2.5. Y-Maze Test

The Y-maze apparatus (Jeung Do B&P, Seoul, Republic of Korea) was made of a black acrylic plate and had three identical arms (35 cm × 3 cm × 15 cm) at 120° angles from one another. Each mouse was placed at the end of the arm and allowed to explore freely for 8 min. During exploration, the number of arm entries and sequences was recorded. Entries were counted only when all four limbs of the mice were contained within the arm. The alternation was considered as three consecutive entries (i.e., ABC, BCA, and BBC), and the alternation score was defined when the mice entered all three arms (i.e., ABC, CBA, or BCA but not ABA). The spontaneous alternation (%) was calculated as shown in the following equation:

$$\mathrm{Spontaneous}\;\mathrm{althernation}\;\left(\%\right)=\left[\frac{\mathrm{alternation}\;\mathrm{score}}{\mathrm{total}\;\mathrm{arm}\;\mathrm{entry}\;\mathrm{numbers}\;-2}\right]\times 100$$

After the test, the apparatus was cleaned with 70% ethanol to remove odors and residues.

### 2.6. Passive Avoidance Test (PAT)

The PAT apparatus (Jeung Do B&P, Seoul, Republic of Korea) was divided into two compartments, and interaction was made available by a guillotine door placed in the middle. One compartment was illuminated, whereas the other compartment was dark. All the compartments were made of stainless-steel rods at the bottom. During the acquisition trial, the mice were placed in the light compartment for 10 s, and the latency to enter the dark compartment was recorded after the guillotine door was opened. The latency was measured for up to 60 s. In the dark compartment, the mice were delivered to the electric shock (0.5 mA, 3 s) through stainless steel rods and acclimatized for 60 s. After 24 h, the retention trial was performed using the same procedure as the acquisition trial without shock. In the retention trial, latency was considered at a maximum of 300 s.

### 2.7. Preparation of Brain Sample

After the mice were anesthetized with ketamine and xylazine, the brains were immediately removed. Next, the hippocampus was isolated and homogenized in pH 7.4 phosphate buffer or RIPA buffer (10% *w*/*v*). The lysate was centrifuged at 10,000 rpm for 15 min. The supernatant was obtained and stored at −80 °C for Western blotting.

### 2.8. Measurement of Oxidative Stress

The malondialdehyde (MDA) levels in the hippocampus were measured using a modified thiobarbituric acid reactive substances assay, as described in a previous study [41]. In brief, the hippocampus was isolated and homogenized in PBS. The lysate was centrifuged at 10,000 rpm for 15 min. The supernatant of hippocampus was mixed with 8.1% sodium dodecyl sulfate, 20% acetic acid, and 0.8% thiobarbituric acid. Mixtures were boiled for 1 h at 95 °C and then cooled to terminate the reaction. The supernatants were collected by centrifugation at 14,000 rpm for 10 min. The absorbance of the supernatant was measured at 532 nm. The glutathione (GSH) levels in the hippocampus were determined using a commercially available assay kit (Cayman Chemical, Ann Arbor, MI, USA) based on the 5,5-dithiobis (2-nitrobenzoic acid) method. The protein concentration was measured using a commercial BCA assay kit (Thermo Scientific, Waltham, MA, USA).

### 2.9. Hematoxylin and Eosin (H&E) Staining

The whole brain was fixed in 4% paraformaldehyde overnight at 4 °C. After dehydration, the brains were paraffinized and slashed using a microtome (Leica RM 2255; Leica, Wetzlar, Germany). The sections were cut on coronal parts from bregma −1.7 to bregma −2.1 and were 3 μm thick. Subsequently, tissues were deparaffinized with xylene and hydrated with ethanol. After washing, the tissues were stained with Mayer’s H&E-stained tissues and were scanned using the 3D Histech Panoramic 250 Flash II scanner (3DHISTECH, Budapest, Hungary). The digital images were viewed using the 3DHISTECH Panoramic Viewer (3DHISTECH, Budapest, Hungary). Different microscopic fields in each tissue were analyzed using ImageJ software (version 1.53; NIH, Bethesda, MD, USA). For the quantification of total and damaged cells, we calculated three different fields (300 × 220 μm/field) per tissue and used the average number of cells for further calculations.

### 2.10. Cell Culture

The HT-22 cell, a mouse hippocampal neuronal cell line, was kindly provided by Prof. Hyun Jin Choi (CHA University, Seongnam, Republic of Korea), which was cultured in a high-glucose Dulbecco’s modified Eagle’s medium (Welgene, Gyeongsan, Republic of Korea) containing 10% heat-inactivated fetal bovine serum, 100 U/mL penicillin, and 100 μg/mL streptomycin solution (Gibco, Grand Island, NY, USA). Cells were maintained at 37 °C in a 5% CO_2_ humidified incubator.

### 2.11. Cell Viability Assay

3-(4,5-dimethylthiazol-2-yl)-2, 5-diphenyltetrazolium bromide (MTT) assay was used to evaluate cell viability. HT-22 cells were seeded into 96-well plates at a density of 5 × 10^3^ cells/well for 24 h. Then, the cells were incubated with or without FPF (3, 10, and 30 μg/mL), Trolox (50 μM), and ANA12 (10 μM) for 1 h in serum-free medium. The cells were treated with glutamate (10 mM) for 24 h. The MTT reagent (5 mg/mL) was added to the cells for 4 h, and formazan crystals were dissolved in DMSO. Cell viability was evaluated by measuring absorbance at 540 nm using a microplate reader (Bio-Tek Instruments Inc., Winooski, VT, USA). FPF and glutamate were dissolved in phosphate buffer. Trolox and ANA12 were dissolved in DMSO, and treated concentrations included 0.5% DMSO.

### 2.12. Measurement of ROS

The production of ROS was confirmed through the H_2_DCFDA staining. HT-22 cells were seeded at 2.5 × 10^5^ cells/well in 6-well plates for 24 h. After treatment of FPF (10 μg/mL) or Trolox (50 μM), the cells were treated with glutamate (10 mM) for 8 h. Following this, the cells were incubated with 10 µM H_2_DCFDA for 15 min at 37 °C. Cells were washed with phosphate buffer and resuspended to be measured for FACSAria III flow cytometer (BD Biosciences, San Jose, CA, USA). Fluorescence intensity was detected at an excitation wavelength of 488 nm and an emission wavelength of 530 nm.

### 2.13. Cell Preparation

Cultured HT-22 cells were seeded at 3 × 10^5^ cells/well in 6-well plates for 24 h. The treated cells were washed with 1× PBS and then lysed in RIPA buffer containing a protease inhibitor cocktail. The lysate was centrifuged at 10,000× *g* rpm for 15 min at 4 °C. After collecting only the supernatant, the protein concentration was quantified using a commercial BCA assay kit (Thermo Scientific, Waltham, MA, USA).

### 2.14. Western Blot Analysis

To separate proteins, proteins were loaded onto SDS-PAGE gels, and electrophoresis was conducted. Proteins were transferred onto polyvinylidene fluoride membranes and blocked with 5% skim milk. The anti-BDNF, anti-TrkB, anti-pERK, anti-ERK, anti-pCREB, anti-CREB antibodies (1:1000; Cell Signaling), anti-pTrkB (1:1000; Abcam), and anti-GAPDH (1:5000, Cell Signaling) were incubated at 4 °C overnight. Following this, the membrane was incubated with horseradish peroxidase-conjugated secondary antibodies at room temperature for 1 h. Protein band signals were visualized using the ECL Western blotting detection system (Amersham Biosciences, Pittsburgh, PA, USA) and obtained using an Amersham™ ImageQuant 800 (Cytiva Life Sciences, Marlborough, MA, USA, formerly GE Healthcare Life Sciences). Blot intensity was analyzed by densitometric analysis using ImageJ software (version 1.53, NIH, Bethesda, MD, USA).

### 2.15. DPPH Radical-Scavenging Assay

The radical-scavenging activity of FPF was determined by using DPPH methods as previously described [42]. Various concentrations of FPF (1 to 300 μg/mL) and 250 µM DPPH solution were added to wells in 100 µL, respectively. The DPPH was dissolved in methanol. After reacting for 30 min, the absorbance was measured at 517 nm. The DPPH radical scavenging activity (%) was calculated using the following equation:

$$\mathrm{DPPH}\;\mathrm{radical}\;\mathrm{scavenging}\;\mathrm{activity}\;\left(\%\right)=\frac{\left(A0-\;A1\right)}{A0}\times 100$$

where A0 is the control group and A1 is the extract/standard.

### 2.16. ABTS Radical-Scavenging Assay

The ABTS radical-scavenging activity of FPF was measured as previously described [43]. Approximately 14 mM ABTS solution and 4.9 mM potassium persulfate were mixed in equal volumes and incubated at room temperature in the dark overnight. ABTS solution and potassium persulfate were dissolved in distilled water, and the final concentration of the mixture was 7 mM ABTS and 2.45 mM potassium persulfate. Prior to use, the mixture was diluted in ethanol to an absorbance of 0.70 ± 0.05 at 734 nm. Following this, 180 µL of diluted mixture was incubated with 20 µL of FPF (1 to 1000 μg/mL) at room temperature in the dark for 10 min. Absorbance was measured at 734 nm. The ABTS radical scavenging activity (%) was calculated using the following equation:

$$\mathrm{ABTS}\;\mathrm{radical}\;\mathrm{scavenging}\;\mathrm{activity}\;\left(\%\right)=\frac{\left(A0-\;A1\right)}{A0}\times 100$$

where A0 is the control group and A1 is the extract/standard.

### 2.17. Statistical Analysis

GraphPad Prism 8.0.2 (GraphPad Software, Inc., La Jolla, CA, USA) was used for data analysis and graph drawing. All data are expressed as mean ± standard error of the mean (SEM), and statistical significance was compared using one-way analysis of variance (ANOVA) with Dunnett’s post hoc test for unpaired observations between the two groups. For all analyses, statistical significance was set at *p* < 0.05.

## 3. Results

### 3.1. FPF Treatment Improves SD-Induced Cognitive Impairment in Mice

To investigate whether FPF improved cognitive impairment, we assessed cognitive performance using the Y-maze test and PAT in sleep-deprived mice. The mice exposed to SD exhibited cognitive impairment compared to the control mice, which were evident from the reduction in spontaneous alterations in the Y-maze test (50.60 ± 3.86%, *p* < 0.01) and latency time in the PAT (59.10 ± 7.52 s, *p* < 0.01). To determine the optimal concentration of FPF, we first tested its cognitive improvement effects using a log scale of 10, 30, and 100 mg/kg. The results showed no effect at 30 mg/kg, while a significant improvement was observed at 100 mg/kg. To identify the minimum effective dose, we tested 50 mg/kg, which lies between 30 and 100 mg/kg, and for the maximum effective dose, we used 150 mg/kg. The results indicated a partial effect at 50 mg/kg, while 150 mg/kg showed a similar or slightly unstable pattern compared to 100 mg/kg. Therefore, we selected a range of 30 to 100 mg/kg to explore the cognitive improvement effects. In the Y-maze test, FPF significantly improved cognitive impairment at 100 mg/kg (72.38 ± 2.88%, *p* < 0.01, Figure 1c). On the other hand, PAT results showed significant improving effects on cognitive impairment at similar levels at doses of 50 and 100 mg/kg of FPF (50 mg/kg, 164.80 ± 21.94 s; 100 mg/kg, 163.20 ± 29.02 s, *p* < 0.01, Figure 1e). Therefore, all subsequent experiments were conducted using the FPF 100 mg/kg dose, which showed the most significant cognitive improvement. On the other hand, there were no differences between the groups in the total arm entries in the Y-maze test or the latency time of the acquisition trial in the PAT (Figure 1b,d). Based on this result, as FPF was administered concurrently during SD, it suggests that 100 mg/kg FPF has both therapeutic and preventive effects against SD-induced cognitive impairment.

### 3.2. FPF Treatment Suppresses SD-Induced Hippocampal Oxidative Stress in Mice

**Figure 2:**
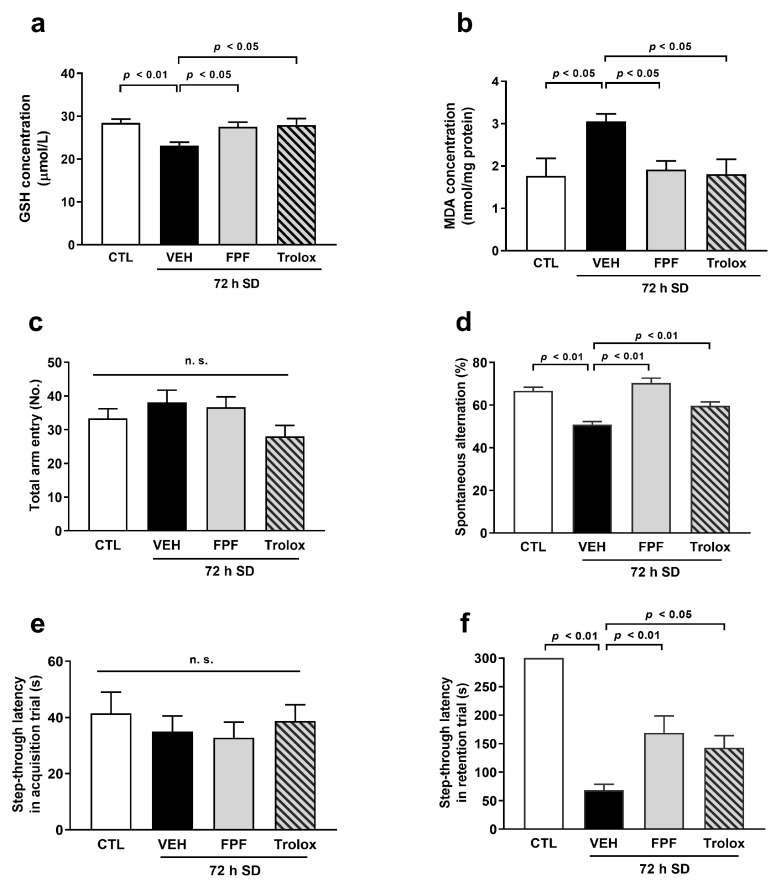
Effect of FPF on oxidative stress in sleep-deprived mice. FPF (100 mg/kg, p.o) and Trolox (50 mg/kg, p.o) were orally administered to sleep-deprived mice. The levels of (**a**) GSH (one-way ANOVA: F_(3, 27)_ = 6.62, *p* = 0.0017) and (**b**) MDA (one-way ANOVA: F_(3, 26)_ = 4.398, *p* = 0.0125) in the hippocampus. (**c**) The total arm entries (one-way ANOVA: F_(3, 31)_ = 1.475, *p* = 0.1347) and (**d**) the spontaneous alternation (one-way ANOVA: F_(3, 31)_ = 12.79, *p* < 0.0001) in the Y-maze test. (**e**) The step-through latency in acquisition trial (one-way ANOVA: F_(3, 36)_ = 0.3843, *p* = 0.7649) and (**f**) the step-through latency in retention trial (one-way ANOVA: F_(3, 36)_ = 26.17, *p* < 0.0001) of the PAT. Data are presented as the mean ± SEM (*n* ≤ 10). CTL, control; VEH, vehicle; SD, sleep deprivation; n.s., not significant; ANOVA, analysis of variance; SEM, standard error of mean.

We evaluated the effects of FPF on oxidative stress in the hippocampus of sleep-deprived mice. Hippocampal tissues were collected immediately after the behavioral test, and GSH and MDA levels were measured. The sleep-deprived mice showed significantly decreased GSH levels (22.93 ± 0.73 μmol/L, *p* < 0.01) and increased MDA levels (3.05 ± 0.18 nmol/mg protein, *p* < 0.05) compared to the control mice. However, FPF (100 mg/kg) treatment remarkably restored the levels of GSH (27.54 ± 1.07 μmol/L, *p* < 0.05) and MDA (1.92 ± 0.21 nmol/mg protein, *p* < 0.05) (Figure 2a,b). Moreover, we evaluated the effects of Trolox, a representative radical scavenger, on decreased GSH, increased MDA levels, and cognitive impairment in sleep-deprived mice. The Trolox (50 mg/kg) showed antioxidant effect on oxidative stress in GSH (27.96 ± 1.48 μmol/L, *p* < 0.05) and MDA levels (1.81 ± 0.35 nmol/mg protein, *p* < 0.05) and improving effect on cognitive impairment in both the Y-maze test (59.63 ± 1.87, *p* < 0.01) and PAT (142.80 ± 21.28, *p* < 0.05) (Figure 2a–d). This result indicates that the antioxidant effect of FPF can improve and prevent cognitive impairment induced by SD.

### 3.3. FPF Treatment Increases BDNF Expression and TrkB Antagonist Abolishes the Effect of FPF on Cognitive Impairment in Sleep-Deprived Mice

**Figure 3:**
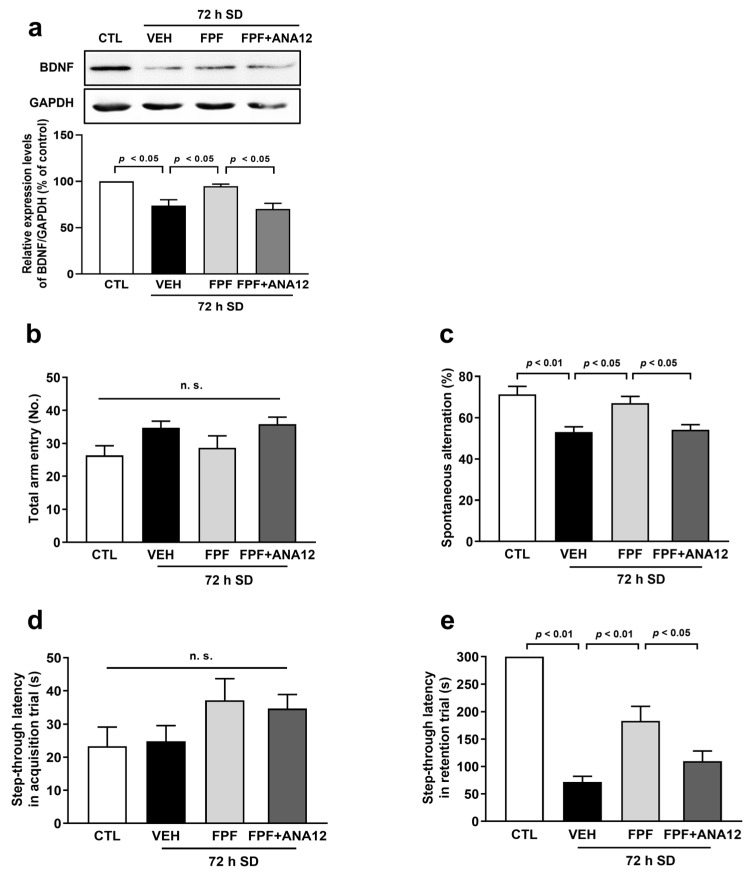
Roles of BDNF in the improving effect of FPF on cognitive impairment induced by SD. Mice were administered ANA12 (0.5 mg/kg, i.p) 15 min before the administration of FPF (100 mg/kg, p.o). (**a**) Expression level of BDNF (one-way ANOVA: F_(3, 8)_ = 10.89, *p* = 0.0034) by Western blotting. (Top) Representative Western blot image; (bottom) histogram of relative quantification. (**b**) The total arm entries (one-way ANOVA: F_(3, 35)_ = 2.747, *p* = 0.0575) and (**c**) the spontaneous alternation (one-way ANOVA: F_(3, 35)_ = 9.076, *p* = 0.0001) in the Y-maze test. (**d**) The step-through latency in acquisition trial (one-way ANOVA: F_(3, 36)_ = 1.692, *p* = 0.1861) and (**e**) the step-through latency in retention trial (one-way ANOVA: F_(3, 36)_ = 34.71, *p* < 0.0001) in of the PAT. Data are presented as the mean ± SEM (*n* ≤ 10). CTL, control; VEH, vehicle; SD, sleep deprivation; n.s., not significant; ANOVA, analysis of variance; SEM, standard error of mean.

We evaluated whether FPF could increase the BDNF expression in the hippocampus and investigated the involvement of BDNF signaling on the improving effect of FPF on cognitive impairment by using ANA12, a TrkB receptor antagonist. Relative to the control mice, the sleep-deprived mice had a lower expression level of BDNF in the hippocampus (73.98 ± 6.25%, *p* < 0.05), which was increased by FPF (100 mg/kg) treatment (94.89 ± 2.23%, *p* < 0.05). In addition, administration of ANA12 (0.5 mg/kg) significantly abolished the effect of FPF on the BDNF expression (70.24 ± 6.09%, *p* < 0.05) (Figure 3a). Moreover, ANA12 also abolished the effect of FPF on the cognitive impairment in both the Y-maze test (54.14 ± 2.45%; *p* < 0.05) and PAT (109.6 ± 18.78 s, *p* < 0.05) (Figure 3c,e).

### 3.4. FPF Treatment Increases Phosphorylation Levels of TrkB/ERK/CREB in Sleep-Deprived Mice

**Figure 4:**
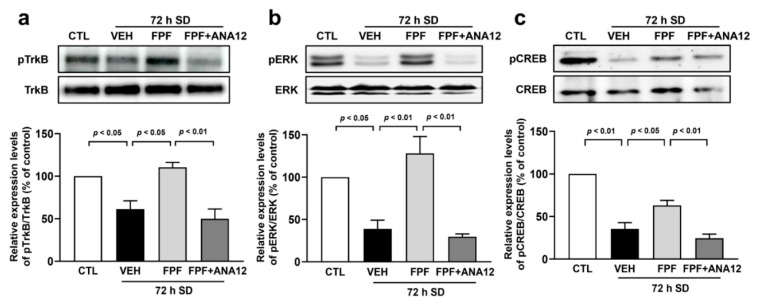
Effect of FPF on the phosphorylation levels of TrkB/ERK/CREB in the hippocampus of sleep-deprived mice. Mice were administered ANA12 (0.5 mg/kg, i.p) 15 min before the administration of FPF (100 mg/kg, p.o). Expression levels of (**a**) pTrkB/TrkB (one-way ANOVA: F_(3, 8)_ = 13.15, *p* = 0.0018), (**b**) pERK/ERK (one-way ANOVA: F_(3, 8)_ = 17.97, *p* = 0.0006) and (**c**) pCREB/CREB (one-way ANOVA: F_(3, 8)_ = 41.10, *p* < 0.0001) by Western blotting. (Top) Representative Western blot image; (bottom) histogram of relative quantification. Data are presented as the mean ± SEM (*n* = 3). CTL, control; VEH, vehicle; SD, sleep deprivation; FPF, fermented *Perilla frutescens*; ANA12, *N*-[2-[[(hexahydro-2-oxo-1H-azepin-3-yl)amino]carbonyl]phenyl]-benzo[b]thiophene-2-carboxamide; TrkB, tropomyosin receptor kinase B; ERK, extracellular signal-regulated kinase; CREB, cAMP response element-binding protein; GAPDH, glyceraldehyde-3-phosphate dehydrogenase.

To understand the mechanisms underlying BDNF signaling, we investigated the BDNF signaling-related molecules. The total and phosphorylated protein levels of TrkB, ERK, and CREB in the hippocampus were evaluated using Western blotting. The quantification of each phosphorylated protein was corrected based on the total protein amount. In sleep-deprived mice, phosphorylated levels of TrkB (61.36 ± 9.71%, *p* < 0.05), ERK (38.89 ± 10.31%, *p* < 0.05), and CREB (35.55 ± 7.31%, *p* < 0.01) were significantly lower than in control mice. However, these reductions were attenuated by FPF (100 mg/kg) treatment (TrkB, 110.54 ± 5.63%; ERK, 128.21 ± 19.71%; CREB, 63.08 ± 5.80%, *p* < 0.05). Furthermore, ANA12 (0.5 mg/kg) co-treatment with FPF (100 mg/kg) abrogated the change induced by FPF treatment on the phosphorylated levels of TrkB (49.79 ± 11.70%, *p* < 0.01), ERK (29.72 ± 3.23, *p* < 0.01), and CREB (24.45 ± 4.82%, *p* < 0.01) (Figure 4). These results suggest that BDNF signaling may play an important role in the improvement and prevention effects of FPF on cognitive impairment induced by SD.

### 3.5. FPF Treatment Reduces SD-Induced Hippocampal Neuronal Damage in Mice

**Figure 5:**
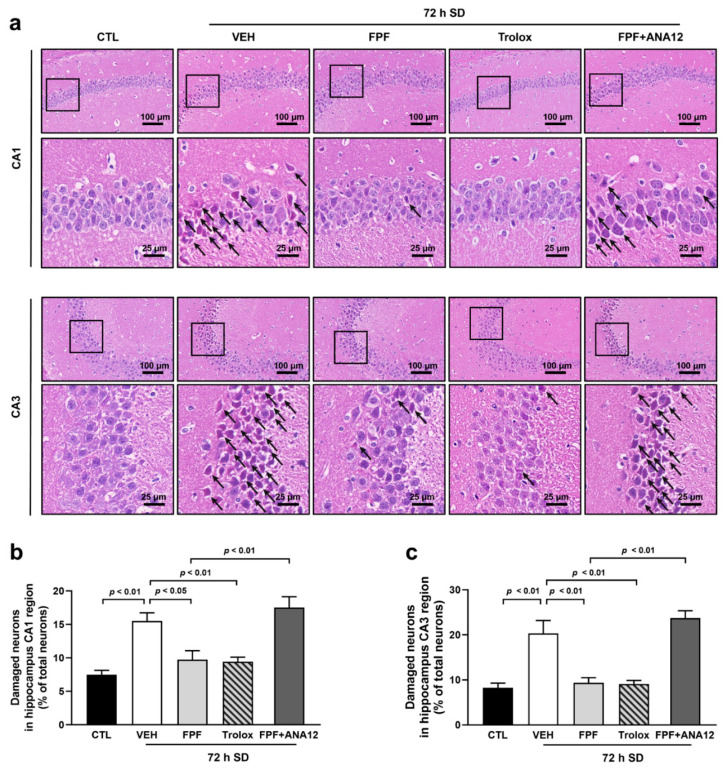
Effect of FPF on neuronal damage in the hippocampus of sleep-deprived mice. (**a**) H&E staining of CA1 and CA3 regions in the hippocampus. The enlarged regions are within respective rectangular boxes. Scale bar = 100 μm or 25 μm. Black allows indicated pyknotic neurons. The percentage of damaged neurons in (**b**) CA1 (one-way ANOVA: F_(4, 19)_ = 14.57, *p* < 0.0001) and (**c**) CA3 (one-way ANOVA: F_(4, 17)_ = 21.14, *p* < 0.0001) regions relative to the total neurons for each group. Black arrows, pathological changes in damaged neurons. Data are presented as the mean ± SEM (*n* ≤ 5). CTL, control; VEH, vehicle; SD, sleep deprivation; FPF, fermented *Perilla frutescens*; Trolox, 6-Hydroxy-2,5,7,8-tetramethylchroman-2-carboxylic acid; ANA12, *N*-[2-[[(hexahydro-2-oxo-1H-azepin-3-yl)amino]carbonyl]phenyl]-benzo[b]thiophene-2-carboxamide; CA1, cornu ammonis1; CA3, cornu ammonis3.

Hippocampal injury is the major cause of cognitive impairment. Therefore, we evaluated morphological changes in hippocampal neurons to determine whether FPF attenuated neuronal damage. SD induces pathological change in the cornu ammonis1 (CA1) and cornu ammonis3 (CA3) regions of the hippocampus. Compared with control mice, sleep-deprived mice appeared loosely arranged, and pathological changes, including edema, shrunken neurons, and darkly stained pyknotic nuclei, were observed (Figure 5a). In sleep-deprived mice, the percentage of damaged neurons values in total neurons was significantly increased in both the CA1 (15.52 ± 1.23% of the total neurons, *p* < 0.01) and CA3 (20.35 ± 2.84% of the total neurons, *p* < 0.01) regions compared with control mice. In contrast, FPF (100 mg/kg) treatment showed recovered pathological change of hippocampal neurons and remarkably decreased the percentage of damaged neurons in both the CA1 (9.75 ± 1.33% of the total neurons, *p* < 0.05) and CA3 (9.37 ± 1.11% of the total neurons, *p* < 0.01) regions (Figure 5b,c). Moreover, the administration of Trolox (50 mg/kg) showed neuroprotective effects against SD-induced damage, and co-treatment of ANA12 (0.5 mg/kg) and FPF (100 mg/kg) increased abnormally shaped neurons and increased the percentage of damaged neurons in CA1 (17.53 ± 1.62% of total, *p* < 0.01) and CA3 (ANA12, 23.73 ± 1.64% of total, *p* < 0.01) regions compared with that in the FPF-treated mice.

### 3.6. FPF Treatment Protects Glutamate-Induced Cell Death in HT-22 Cells

**Figure 6:**
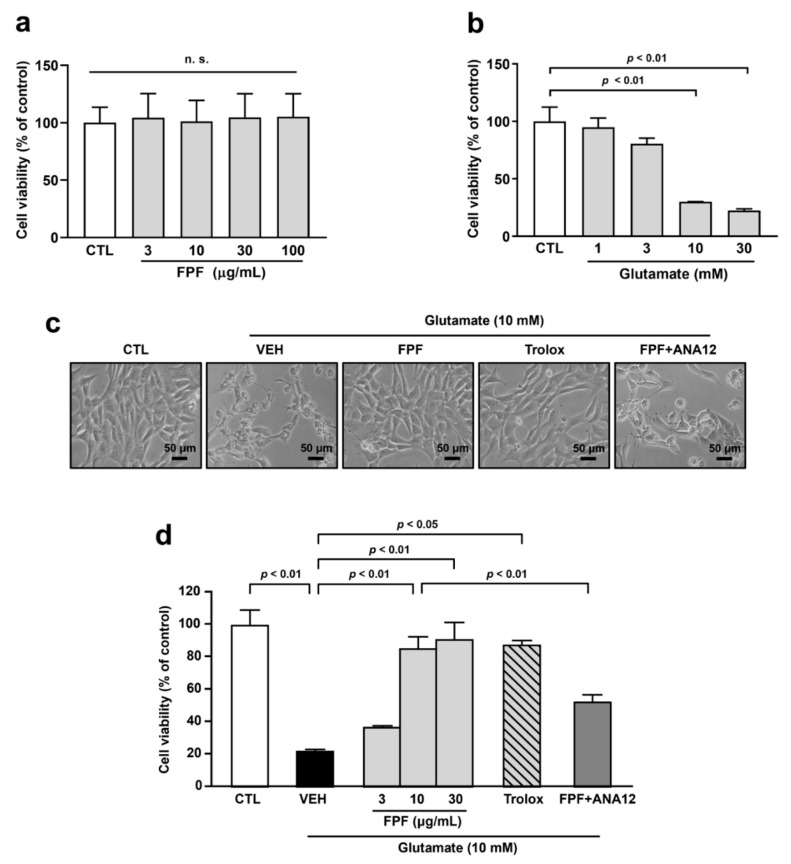
Effect of FPF on glutamate-induced cell death in HT-22 cells. (**a**) The cytotoxicity of FPF (3, 10, 30 and 100 μg/mL) on HT-22 cells (one-way ANOVA: F_(4, 10)_ = 0.01506, *p* = 0.9995). (**b**) The cytotoxicity of glutamate (1, 3, 10, and 30 mM) on HT-22 cells (one-way ANOVA: F_(4, 10)_ = 27.14, *p* < 0.001). Cells were pre-treated with FPF (3, 10 and 30 μg/mL) or Trolox (50 μM) for 1 h. ANA12 (10 μM) was co-treated with 10 μg/mL of FPF. Then, glutamate (10 mM) was treated for 24 h, following cell viability was determined by MTT assay. (**c**) Morphologies of cells. Scale bar = 50 μm. (**d**) Quantitative analysis of cell viability (one-way ANOVA: F_(6, 14)_ = 24.26, *p* < 0.0001). Data are presented as the mean ± SEM (*n* = 3). CTL, control; VEH, vehicle; FPF, fermented *Perilla frutescens*; Trolox, 6-Hydroxy-2,5,7,8-tetramethylchroman-2-carboxylic acid; ANA12, *N*-[2-[[(hexahydro-2-oxo-1H-azepin-3-yl)amino]carbonyl]phenyl]-benzo[b]thiophene-2-carboxamide; MTT, 3-(4,5-dimethylthiazol-2-yl)-2,5-diphenyltetrazolium bromide.

To support neuroprotective effects of FPF in the hippocampus, we did further investigations using HT-22 cells. We found that FPF had no cytotoxicity effects within a concentration range of 3 to 100 μg/mL (Figure 6a). We tested various concentrations of glutamate to determine the optimal concentration for glutamate toxicity. Since 10 mM of glutamate significantly caused less than 50% cell viability (30.17 ± 0.17%, *p* < 0.01), the 10 mM concentration of glutamate was selected for further experiments (Figure 6b). Treatment of FPF (3, 10, and 30 μg/mL) increased cell viability in a concentration-dependent manner against glutamate-induced cytotoxicity (10 μg/mL, 85.65 ± 8.31%; 30 μg/mL, 91.19 ± 12.58%, *p* < 0.01). As no significant difference was observed between 10 μg/mL and 30 μg/mL of FPF, we performed the subsequent experiments using 10 μg/mL of FPF as the lowest effective concentration. Further investigation showed that treatment with Trolox (50 μM) has protective effects against glutamate-induced toxicity (87.83 ± 3.00%, *p* < 0.01). In addition, ANA12 (10 μM) co-treatment with FPF (10 μg/mL) abolished some protective effects of FPF in glutamate-exposed HT-22 cells (52.74 ± 5.32, *p* < 0.05) (Figure 6c,d).

### 3.7. FPF Treatment Reduces Oxidative Stress and Enhances BDNF Expression in Glutamate-Exposed HT-22 Cells

**Figure 7:**
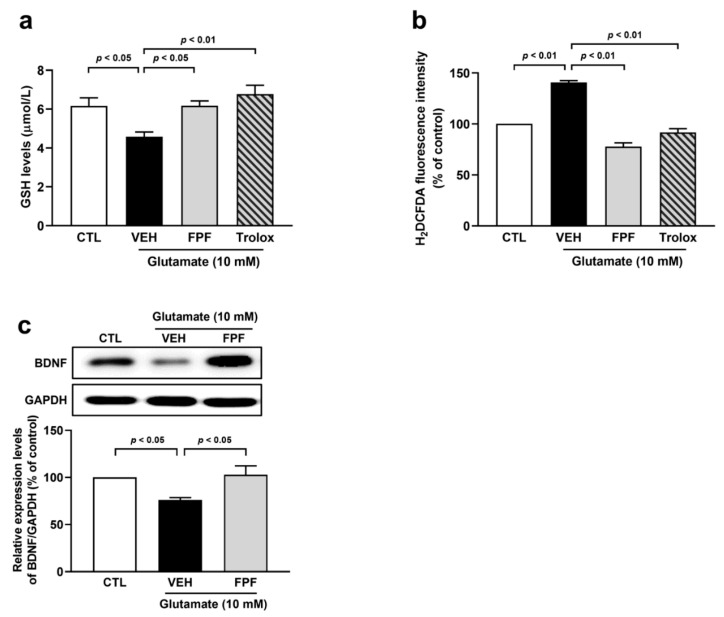
Effect of FPF on oxidative stress and BDNF expression in glutamate-exposed HT-22 cells. Cells were pre-treated with FPF (10 μg/mL) or Trolox (50 μM) for 1 h, following glutamate (10 mM) for 24 h. The levels of intracellular (**a**) GSH (one-way ANOVA: F_(3, 14)_ = 6.519, *p* = 0.0055) and (**b**) ROS (one-way ANOVA: F_(3, 8)_ = 99.04, *p* < 0.0001). (**c**) Expression levels of BDNF (one-way ANOVA: F_(2, 9)_ = 6.671, *p* = 0.0167) by Western blotting. (Top) Representative Western blot image; (bottom) histogram of relative quantification. Data are presented as the mean ± SEM (*n* ≤ 5). CTL, control; VEH, vehicle; FPF, fermented *Perilla frutescens*; Trolox, 6-Hydroxy-2,5,7,8-tetramethylchroman-2-carboxylic acid; H_2_DCFDA, 2,7-dichlorodihydro fluorescent diacetate; ROS, reactive oxygen species; BDNF, brain-derived neurotrophic factor; GAPDH, glyceraldehyde-3-phosphate dehydrogenase.

To confirm these in vivo results in mice, we investigated the effects of FPF on glutamate-induced oxidative stress in HT-22 cells. Glutamate significantly decreased the GSH levels (4.58 ± 0.24 μmol/L, *p* < 0.05), which increased through FPF (10 μg/mL) and Trolox (50 μM) treatment (FPF, 6.17 ± 0.25 μmol/L; Trolox, 6.78 ± 0.45 μmol/L, *p* < 0.01) (Figure 7a). Moreover, FPF (10 μg/mL) and Trolox (50 μM) treatment inhibited the increased accumulation of ROS (FPF, 77.85 ± 3.57%; Trolox, 91.63 ± 3.72% *p* < 0.01) induced by glutamate (Figure 7b). We found that glutamate induced a reduction in BDNF protein levels (76.01 ± 2.55, *p* < 0.05). Meanwhile, FPF (10 μg/mL) caused a significant increase in the expression levels of BDNF (102.76 ± 9.53, *p* < 0.05) (Figure 7c). These results indicate that FPF demonstrates improvement and preventive effects against glutamate-induced neuronal damage through its antioxidant activity and BDNF signaling.

### 3.8. Antioxidant Activity of FPF

**Figure 8:**
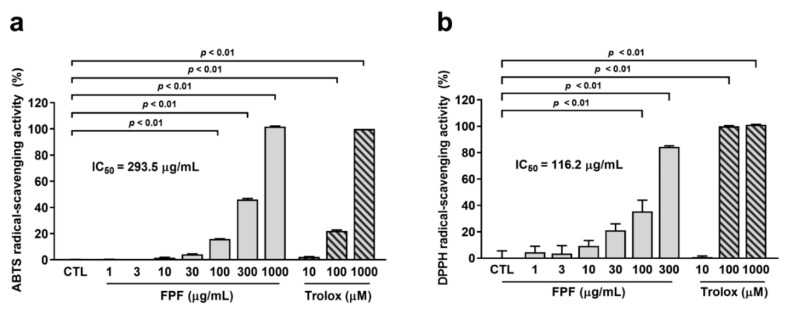
In vitro antioxidant activity of FPF. (**a**) ABTS radical-scavenging assay (one-way ANOVA: F_(7, 31)_ = 9267, *p* < 0.0001) and (**b**) DPPH radical-scavenging assay (one-way ANOVA: F_(9, 40)_ = 88.60, *p <* 0.0001). The CTL group was used as the vehicle of FPF. Data are presented as the mean ± SEM (*n* = 5). CTL, control; FPF, fermented *Perilla frutescens*; Trolox, 6-Hydroxy-2,5,7,8-tetramethylchroman-2-carboxylic acid; ABTS, 2,2′-azinobis (3-ethylbenzothiazoline-6-sulfonic acid); DPPH, 2,2-diphenyl-1-picrylhydrazyl; IC_50_; half-maximal inhibitory concentration.

We assessed the radical-scavenging activity of FPF in vitro. Trolox was used as a positive control. ABTS radical-scavenging ability ranged from 100 to 1000 μg/mL in FPF (100 μg/mL FPF, 15.90 ± 0.16%; 300 μg/mL FPF, 46.07 ± 0.75; 1000 μg/mL FPF, 101.72 ± 0.41, *p* < 0.01) (Figure 8a). Meanwhile, DPPH radical-scavenging ability ranged from 100 to 300 μg/mL in FPF (100 μg/mL FPF, 35.43 ± 8.58%; 300 μg/mL FPF, 84.44 ± 0.70%, *p* < 0.01) (Figure 8b). FPF showed IC_50_ values of 293.5 μg/mL and 116.2 μg/mL in ABTS and DPPH assays, respectively. The radical-scavenging ability of FPF was more prominent in the DPPH assay than in the ABTS radical-scavenging assay.

## 4. Discussion

In this study, we found that FPF treatment improved cognitive impairment and reversed hippocampal neuronal damage in sleep-deprived mice. Furthermore, the potential mechanisms of action of FPF are associated with its antioxidant activity and increased BDNF expression. FPF reduced ROS accumulation and MDA levels by increasing GSH levels during hippocampal oxidative stress. Moreover, the antioxidant activity of FPF includes radical-scavenging activity. As another mechanism, FPF treatment increased BDNF expression and phosphorylation of TrkB, ERK, and CREB. The BDNF/TrkB/ERK/CREB signaling pathway is associated with the neuroprotective and cognitive ameliorating effects of FPF. Taken together, these results indicate that FPF not only protects against oxidative stress but also increases BDNF expression, thereby ameliorating SD-induced cognitive impairment. To the best of our knowledge, this is the first study to report the effects of FPF on SD-induced cognitive impairment.

Cognitive function has been conceptualized to include memory, attention, and executive function [44]. Memory is one of the most complex concepts in cognitive function and comprises three distinct processes, i.e., encoding, consolidation, and information retrieval [44,45]. Memory encoding and retrieval occur during waking, but the consolidation of newly acquired information is facilitated through unique neuromodulatory activity during sleep [46]. Memory encoded during wakefulness is associated with episodic representations in the hippocampus as a hub [45]. Non-rapid movement (NREM) sleep mediates the redistribution of hippocampus-dependent memories into long-lasting storage sites, and subsequent rapid eye movement (REM) sleep supports synaptic consolidation through the local upregulation of plasticity-related genes and proteins [47,48]. Indeed, according to clinical studies, SD impairs emotional working memory and increases the formation of false memories [49]. Moreover, SD impairs cognitive function and aggravates AD neuropathology [50]. Therefore, sleep is crucial for cognitive function. In the present study, we used a modified multi-platform method to disturb sleep. Our results showed that cognitive impairment caused by 72 h SD could be improved by FPF through a variety of behavioral experiments capable of assessing spatial and contextual memory [51,52]. Because both spatial and contextual memories are considered to be hippocampus-dependent, it may be suggested that FPF have a positive effect on SD-induced hippocampal dysfunction [53,54]. The hippocampus is one of the major brain regions involved in the optimization of learning and memory and the consolidation of declarative memory [13]. Rats with bilateral hippocampal lesions exhibited impaired spatial and recognition memory [55]. Also, the volume of the hippocampus is smaller in patients with insomnia than in normal individuals, and these results are associated with cognitive impairment [56]. The administration of FPF decreased neuronal loss and maintained neuronal morphology in the hippocampal CA1 and CA3 regions. The CA1 region is responsible for the collection of spatial memory data and critically affects hippocampus-dependent memory [57,58]. In addition, because the CA3 region has many internal connectivities relative to other regions, it has been reported to play a strong role in spatial and episodic memory [59]. Considering the importance of the hippocampus in cognitive function, we can assume that the ameliorative effect of FPF on cognitive impairment can be attributed to its neuronal protective effects.

The major proposed mechanisms for SD-induced cognitive impairment are oxidative stress mediating the hippocampal injury [20,57]. The brain is organized into a highly developed mitochondrial and synaptic network responsible for maintaining brain function, which leads to high levels of metabolic products such as ROS and reactive nitrogen species (RNS). Accumulated ROS and RNS can induce brain damage in sleep-deprived mice [60]. In addition, SD may downregulate immune responses by increasing pro-inflammatory signaling and ROS levels by inhibiting antioxidant defense mechanisms [61,62]. Therefore, the use of antioxidants may decrease oxidative stress in the hippocampus and improve cognitive impairment induced by SD [40]. Here, we found that treatment with FPF protected the hippocampus from SD-induced oxidative stress by enhancing GSH levels. GSH is a major nonenzymatic antioxidant and the most important and abundant thiol-containing peptide in the brain [63]. Moreover, measurement of GSH is important because it can reflect the balance between oxidative stress and antioxidant systems [64]. GSH plays a role in the detoxification of hydrogen peroxide (H_2_O_2_) as an electron donor via glutathione peroxidase. Accumulated radicals attack lipids containing carbon–carbon double bond(s), such as polyunsaturated fatty acids, resulting in the production of cytotoxic aldehydes such as MDA. In general, MDA is used as a biomarker for oxidative stress [65,66]. In the present study, we found that FPF significantly decreased hippocampal MDA levels, which were increased by SD. Taken together, these results indicate that oxidative stress is increased with decreased GSH in the hippocampus by SD, whereas FPF protects the hippocampus by eliminating H_2_O_2_ through an increase in GSH levels. We further evaluated whether FPF has a protective effect on glutamate-induced cell death in HT-22 cells, a mouse hippocampal neuronal cell line as an in vitro model. Glutamate levels were upregulated during wakefulness and REM sleep and downregulated during NREM sleep, thus regulating homeostasis throughout the sleep process [5,67]. Indeed, SD increases glutamate levels in the hippocampus and thalamus [17]. Excessive glutamate levels lead to neuronal excitotoxicity and cell death through mitochondrial dysfunction and ROS accumulation [68]. Moreover, we demonstrated that the low levels of GSH in the hippocampus after SD are the main cause of oxidative stress that causes hippocampal damage. The mechanism of glutamate-induced cytotoxicity to HT-22 cells is primarily reported to be depletion of intracellular GSH levels by blocking the cystine/glutamate antiporter [69,70,71]. Furthermore, previous reports show that the light-chain subunit (xCT) of the cystine/glutamate antiporter reduced by SD in the hippocampus [72]. Therefore, we used glutamate-exposed HT-22 cells as a suitable in vitro model for oxidative toxicity due to GSH depletion after SD. In the present study, we observed that FPF not only has a protective effect on glutamate-induced cell death but also has a protective effect against oxidative stress. These findings indicated that FPF has a protective effect against oxidative stress both in vivo and in vitro. Therefore, we identified the effect of Trolox on cognitive impairment as a positive control. Trolox, a water-soluble analog of vitamin E, acts as a radical scavenger and has been evaluated as a better antioxidant than vitamin E [73,74]. Here, we found that Trolox ameliorated the cognitive impairment caused by SD and had a protective effect against hippocampal neuronal cell death in both in vivo and in vitro models.

Several studies have reported that the cognitive impairment induced by SD is related to a reduction in BDNF levels in the hippocampus [20,21]. Previously, we reported that SD decreases BDNF expression, and FPF has antidepressant-like efficacy by upregulating BDNF expression in the hippocampus [38]. However, evidence strongly implicates the role of BDNF in cognitive function, as well as its antidepressant effects [75,76,77]. BDNF expression was elevated in the hippocampus after learning [78]. BDNF expression was detected in all brain regions, with the highest levels found in the hippocampus [79]. The acts of BDNF through the TrkB receptor promote three major downstream signaling pathways, like mitogen-activated protein kinase, phospholipase C-γ, and phosphatidylinositol 3-kinase [80]. Among these, the BDNF/TrkB/ERK pathway is a well-characterized signaling pathway that induces the phosphorylation of CREB [80,81]. CREB is a transcription factor responsible for the transcriptional activity of genes involved in neural function such as neuronal development, plasticity, and protection [82]. In addition, CREB contains BDNF as a downstream target of transcription [83]. Here, we revealed that FPF alleviated SD-induced reductions in hippocampal BDNF expression levels, and this was accompanied by the phosphorylation of TrkB, ERK, and CREB. Therefore, to delve into the role of BDNF signaling in the mechanism by which FPF improves cognitive impairment induced by SD, a TrkB receptor antagonist was blocked. In an in vivo test, the inhibition of the TrkB receptor not only affected molecular changes but also reversed the improving effect of FPF on SD-induced cognitive impairment in mice. In addition, the inhibition of TrkB receptor abolished the protective effects of FPF against hippocampal neuronal damage both in vivo and in vitro. These results imply that FPF is probably involved in the cognitive impairment induced by SD through activation of the BDNF/TrkB/ERK/CREB signaling pathway in the hippocampus under SD-induced pathological conditions.

We have previously established that FPF has a beneficial effect on both sleep and stress [38,84]. This suggests that FPF not only improves cognitive function but also exhibits protective effects against various mental disorders. In previous reports, we identified six main components of FPF: uracil, adenine, protocatechuic acid (PCA), luteolin-7-O-diglucuronide (L7dGn), apigenin-7-O-diglucuronide (A7dGn), and luteolin-7-O-glucuronide (L7Gn). Among these, PCA and L7Gn were found to be associated with sleep and stress. Although our study did not directly link these components to sleep deprivation-induced cognitive impairment, it is possible that the improving efficacy of FPF on cognitive impairment may be attributed to the activity of PCA and L7Gn or their combined effects. Indeed, PCA is known to improve cognitive impairment [85], and L7Gn is recognized for its neuroprotective effects [86]. PCA is a phenolic compound known to exhibit significantly enhanced biological activity through fermentation and is reported to cross the blood–brain barrier (BBB), exerting neuroprotective and cognitive impairment-improving effects [87,88]. Specifically, PCA has been reported to enhance DPPH scavenging activity, reduce MDA levels, and increase GSH and SOD levels, ultimately improving cognitive function [89,90]. Therefore, it is likely to play a central role in the effects of FPF. On the other hand, compounds such as L7Gn and A7dGn have a very low probability of crossing the BBB due to their size and ionic properties. However, considering their physicochemical characteristics, alternative mechanisms, such as carrier-mediated transport, could facilitate their passage through the BBB. Thus, further research is needed to identify the specific components of FPF that primarily mediate its effects in alleviating cognitive impairment caused by SD. Additionally, this study has several more limitations. Although the present study demonstrated the antioxidant efficacy of FPF by showing an increase in GSH levels, further studies are needed to clarify how GSH levels are regulated by SD and FPF. The mechanism by which FPF regulates BDNF levels requires further investigation. Furthermore, since this study was conducted using only male mice, future studies should also include a comparison of the cognitive improvement effects of FPF between sexes by assessing its efficacy in female mice.

In conclusion, our study shows that FPF improves cognitive impairment in SD-induced pathological conditions. The underlying mechanism may involve the antioxidant and BDNF signaling pathways, at least in part.

## Data Availability

The original contributions presented in the study are included in the article, further inquiries can be directed to the corresponding author.
